# Early administration of fibrinogen concentrates improves the short‐term outcomes of severe pelvic fracture patients

**DOI:** 10.1002/ams2.268

**Published:** 2017-04-02

**Authors:** Koichi Inokuchi, Makoto Sawano, Koji Yamamoto, Atsushi Yamaguchi, Satoru Sugiyama

**Affiliations:** ^1^ Department of Emergency and Critical Care Medicine Saitama Medical Center Saitama Medical University Kawagoe Saitama Japan; ^2^ Department of Transfusion Medicine and Cell Therapy Saitama Medical Center Saitama Medical University Kawagoe Saitama Japan

**Keywords:** Blood coagulation disorders, fibrinogen, hemorrhage, pelvic bones, trauma

## Abstract

**Aim:**

Hemorrhage from pelvic fracture is a major cause of mortality after blunt trauma. Several studies have suggested that early fibrinogen supplementation improves outcomes of traumatic hemorrhage. Thus, we revised our massive transfusion protocol (MTP) in April 2013 to include early off‐label administration of fibrinogen concentrate. The objective of this study was to evaluate the impact of the revision on the short‐term outcomes of pelvic fracture patients.

**Methods:**

This was a single‐center, retrospective, cohort study. A total of 224 consecutive pelvic fracture patients hospitalized in Saitama Medical Center (Saitama, Japan), 115 before the revision (Group E) and 109 after (Group L), were enrolled. Characteristics of the patients were compared between the groups. Impacts of the revision were evaluated by hazard ratios adjusted for characteristics, injury severity, and coagulation status using Cox's multivariate proportional hazard model. The impact was also evaluated by log–rank test and relative risk of 28‐day mortality between the groups.

**Results:**

The characteristics were equivalent between the groups. The multivariate analysis revealed that the revision of MTP was significantly related to improved survival with an adjusted hazard ratio (95% confidence interval) of 0.45 (0.07–0.97). The log–rank test gave χ^2^‐test values of 5.2 (*P* = 0.022) and 6.7 (*P* = 0.009), and the relative risks were 0.37 (0.15–0.91) and 0.33 (0.13–0.84), in patients with all Injury Severity Scores and Injury Severity Score ≥21, respectively.

**Conclusion:**

The revision of MTP to include aggressive off‐label treatment with fibrinogen concentrate was related to improved short‐term outcomes of severe pelvic fracture patients. However, due to the limitations of the study, the improvement could not be attributed totally to the revision.

## Introduction

Hemorrhage from pelvic fracture is common and one of the major causes of mortality in severe blunt trauma patients. As control of the hemorrhage and prevention of coagulopathy are directly associated with outcomes, the guidelines published over the last two decades intensively discussed the validities of hemostatic methods such as trans‐arterial embolization (TAE), external fixation, and pelvic packing.^1^, ^2^ However, none of the guidelines refer to hematological correction of coagulopathy by recruitment of coagulation factors.

Conventional approaches for massive traumatic hemorrhage based on fluid resuscitation with blood components have been shown to result in persistent coagulopathy and poor outcomes.^3^ Fibrinogen is the first coagulation factor to fall below a critical value during massive bleeding and hemodilution.^4^ An increasing number of studies have reported the limited efficacy of fresh frozen plasma in treating severe hypofibrinogenemia.^5^ The fibrinogen level at admission is reported to be an independent predictor of mortality for trauma patients,^6^ and another study suggested that fibrinogen supplementation improves outcomes for traumatic hemorrhage.^7^

Based on these findings, we revised our massive transfusion protocol for traumatic hemorrhage (MTP) in April 2013 to include early off‐label administration of fibrinogen concentrate. The objective of this study was to evaluate the impact of the revision on the outcomes of pelvic fracture patients.

## Methods

**Figure 1:**
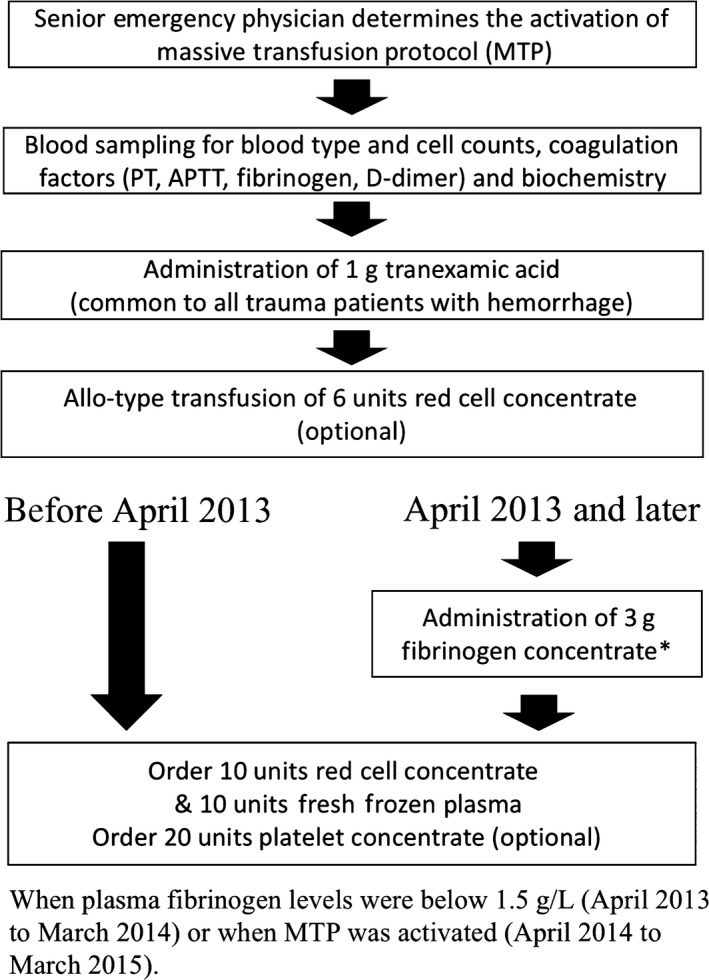
Flow chart to describe the institutional massive transfusion protocol (MTP) for trauma patients. The MTP was revised in April 2013 to include early administration of fibrinogen concentrate. APTT, activated partial thrombin time; PT, prothrombin time.

This was a single‐center, retrospective, cohort study. In 2013, the off‐label use of fibrinogen concentrate (Fibrinogen HT; Japan Blood Products Organization, Tokyo, Japan) for massive traumatic and obstetric hemorrhage was approved by the Institutional Review Board of our hospital. The revision of the MTP to include early off‐label administration of fibrinogen concentrate was substantially put into practice in April 2013. Figure 1 describes the MTP and its revision with a flow chart. In compliance with the revision, we administered 3 g fibrinogen concentrate when plasma fibrinogen levels were below 150 mg/dL (April 2013 to March 2014) or when the MTP was activated (April 2014 to March 2015). Throughout the study period, activation of the MTP was decided by a senior emergency physician who examined the patient on admission.

The study enrolled 224 consecutive patients with pelvic fractures stemming from blunt injuries, who were hospitalized in the Department of Emergency Medicine and Critical Care at Saitama Medical Center (Saitama, Japan) from January 2011 to March 2015. Patients who were in cardiopulmonary arrest on arrival, and those transferred from other hospitals, were excluded. None of the patients had been diagnosed with a disease related to hemostatic disorders. The patients were assigned to two groups: group E, 115 patients who were hospitalized before the revision in April 2013, and group L, 109 patients who were hospitalized later.

Characteristics, injury severities, and status on admission of the patients, which possibly influence their survival, were compared between the groups. They included demographic factors, hospitalization routes and time courses, anticoagulant and antiplatelet medications, abbreviated injury scales, physical and hematological status, and derived scores to predict the survival. The number of blood transfusions within 7 days of admission and implementation of the interventions were also compared between the groups. Derived scores included the Injury Severity Score (ISS), the Revised Trauma Score (RTS), and the Probability of survival (Ps). The interventions included TAE, external fixation, internal fixation, and pelvic packing. If the physical status on the first contact with the patient was not clearly documented, the patients were excluded from the analyses that involved the status or derived scores (RTS and Ps). In the same manner, those patients with missing hematological data were excluded from the analyses concerned. The χ^2^‐test was used for evaluation of intergroup differences in sex, hospitalization routes, medications, allo‐type packed red blood cells transfusion, and implementation of the interventions. Mann–Whitney's *U*‐test was used for others. The significance level was 5% (*P* < 0.05).

The median of ISS in all 224 patients was 21. Based on this threshold, the patients were stratified by ISS (all ISS and ISS ≥ 21). Numbers of patients with ISS ≥ 21 were 60 and 57 in Groups E and L, which included 52 and 44 patients with multiple trauma (AIS > 2 in at least two body regions), respectively. Impacts of the revision and the characteristics, injury severity, and coagulation status on 28‐day survival were evaluated using Cox's multivariate proportional hazard model. The groups (the revision), age, sex, interval between injury and admission, ISS, RTS, and blood hemoglobin concentration, prothrombin time – international normalized ratio, activated partial thrombin time, serum fibrinogen concentration, and platelet count on admission were assigned to the model as explanatory covariates, and 28‐day mortality as the objective variate. Their impact on survival was evaluated in terms of hazard ratios adjusted for other covariates. Impact of the revision on the outcome was also evaluated by the univariate log–rank test between the survival curves, and relative risk of 28‐day mortality between the groups.

All statistical analyses were carried out using R version 3.3.0 (The R Foundation for Statistical Computing, Vienna, Austria) and its packages.^8^, ^9^, ^10^

The off‐label use of the fibrinogen concentrates and the protocol described in this research was approved by the Institutional Review Board and conformed to the provisions of the Declaration of Helsinki (Approval No. 745).

## Results

Tables 1 AND 2 show intergroup comparison of the characteristics, number of blood transfusions, and implementation of the interventions. There was no significant difference between the groups.

**Table 1:** Characteristics of pelvic fracture patients on admission compared between treatment groups

	Group E (*n* = 115)	Group L (*n* = 109)	*P*‐value
Demographic factors, hospitalization route, and time course			
Sex, male : female	48:67	40:69	0.525
Age, years	57 (38.5, 71.5)	59 (42, 71)	0.562
Injury to admission,^a^ min	60 (45.5, 85)	65 (51.5, 110.5)	0.081
Transferred by helicopter	28	23	0.429
Anticoagulant and antiplatelet medications			
Warfarin	3	2	0.952
Aspirin	3	2	0.953
Ticlopidine	2	0	0.590
Abbreviated injury scale			
Head and neck	0 (0, 3)	0 (0, 3)	0.740
Face	0 (0, 0)	0 (0, 0)	0.919
Chest	0 (0, 3)	0 (0, 3)	0.938
Abdomen	0 (0, 2)	0 (0, 2)	0.741
Pelvis and extremities	3 (2, 4)	3 (2, 3)	0.252
Surface	1 (1, 1)	1 (1, 1)	0.933
Physical status on admission (or first contact)			
Glasgow Coma Scale	13 (13, 15)	13 (13, 15)	0.953
Heart rate, b.p.m.	90 (75, 107)	85 (75, 103)	0.357
Systolic blood pressure, mmHg	120 (90, 143)	119 (101, 138)	0.872
Respiratory rate, per min	20 (15, 27)	19 (16, 24)	0.623
No. of patients with missing data^b^	13	11	
Derived scores to predict probabilities of survival			
Injury Severity Score	21 (10, 35)	21 (10, 33)	0.575
Revised Trauma Score	7.55 (6.61, 7.55)	7.55 (7.15, 7.55)	0.070
Probability of survival	0.88 (0.69, 0.97)	0.91 (0.74, 0.98)	0.490
No. of patients with missing data^b^	13	11	
Hematological status on admission			
Hemoglobin concentration, g/dL	12.2 (10.4, 13.4)	12.7 (10.7, 13.9)	0.071
Platelets count, 10^4^/μL	21.1 (15.7, 30.1)	21.5 (16.2, 26.2)	0.549
APTT, s	28.3 (26.0, 33.9)	27.9 (25.1, 31.2)	0.196
PT‐INR	1.17 (1.05, 1.40)	1.14 (1.06, 1.24)	0.186
Fibrinogen concentration, mg/dL	217 (154, 283.5)	214 (150, 254)	0.152
Lactate concentration, mmol/L	3.2 (2.2, 4.8)	3.1 (2.0, 4.2)	0.407
No. of patients with missing data^b^	14	12	

Values represent medians (1st quartile, 3rd quartile) or numbers of patients in Group E (hospitalized before April 2013, before revision of the massive transfusion protocol) and Group L (hospitalized in April 2013 and later, when massive transfusion protocol included early off‐label administration of fibrinogen concentrate).

a Interval between injury and admission.

b Number of the patients with missing data.

APTT, activated partial thrombin time; PT‐INR, prothrombin time – international normalized ratio.

**Table 2:** Number of transfusions and implementation of interventions in pelvic fracture patients compared between treatment groups

	Group E (*n* = 115)	Group L (*n* = 109)	*P*‐value
Number of blood transfusions within 7 days of admission			
Packed red blood cells, units	10 (4, 22)	10 (6, 20)	0.958
Packed red blood cells ≥1 unit^a^	78	68	0.409
Packed red blood cells ≥6 units^b^	55	54	0.297
Allo‐type packed red blood cells^c^	2	3	1.000
Fresh frozen plasma, units	10 (6, 20)	8 (6, 20)	0.685
Platelet concentrate, units	20 (20, 37.5)	20 (20, 20)	0.251
Implementation of interventions			
TAE	36	28	0.764
Injury to TAE, min^d^	184 (156, 220)	178 (146, 211)	0.386
Admission to TAE, min^e^	114 (88.5, 128)	95 (66, 124)	0.279
External fixation	13	14	0.838
Internal fixation	42	43	0.681
Pelvic packing	3	2	1.000

Values represent medians (1st quartile, 3rd quartile) or numbers of patients in Group E (hospitalized before April 2013, before revision of the massive transfusion protocol) and Group L (hospitalized April 2013 and later, when the massive transfusion protocol included early off‐label administration of fibrinogen concentrate).

a Patients who received transfusion of packed red blood cells of any amount.

b Patients who received transfusion of packed red blood cells of 6 units or more.

c Patients who received allo‐type packed red blood cells of 6 units.

d Interval between injury and completion of trans‐arterial embolization (TAE).

e Interval between admission and completion of TAE.

Table 3 shows the results of Cox's multivariate proportional hazard analysis. A “*P* value (rho)” smaller than the significance level (0.05) indicates a violation of the proportional hazard ratio assumption, which is prerequisite of the model. All assigned covariates fulfilled the prerequisite and validated the model. According to the hazard ratios and their confidence intervals, Group L (the revision) and low ISS were significantly related to improved survival.

**Table 3:** Multivariate Cox's proportional hazard analysis of characteristics and treatment of pelvic fracture patients

Patients with all Injury Severity Scores (E : L = 115:117)			
Explanatory covariates	Adjusted hazard ratio^a^	Rho^b^	*P*‐value (rho)^c^
Group L (versus Group E)	0.45 (0.07–0.97)^d^	0.093	0.729
Age, years	1.00 (0.96–1.04)	0.006	0.966
Sex, male versus female	1.13 (0.35–3.62)	−0.023	0.912
Injury to admission, min^e^	1.01 (1.00–1.02)	0.215	0.388
Injury Severity Score	1.06 (1.01–1.11)^d^	0.009	0.965
Revised Trauma Score	0.91 (0.61–1.35)	−0.159	0.456
Hemoglobin concentration, g/dL	0.91 (0.68–1.23)	−0.015	0.937
APTT, s	1.04 (0.95–1.06)	−0.131	0.592
PT‐INR	1.13 (0.77–1.66)	−0.149	0.548
Fibrinogen concentration, mg/dL	1.00 (0.99–1.01)	0.104	0.582
Platelet count, 10^4^/μL	1.00 (0.99–1.01)	−0.441	0.142

a Hazard ratios (95% confidence intervals) adjusted for other covariates.

b Pearson's product‐moment correlation coefficient between scaled Schoenfield residuals and time.

c Probability that “rho” is equivalent to 0 (ratio is independent of time).

d Ratio is significantly smaller or greater than 1 with 95% confidence intervals.

e Interval between injury and admission.

APTT, activated partial thrombin time; E, Group E patients hospitalized before April 2013, before revision of massive transfusion protocol; L, Group L patients, hospitalized April 2013 and later, massive transfusion protocol revised to include early off‐label administration of fibrinogen concentrate; PT‐INR, prothrombin time – international normalized ratio.

**Figure 2:**
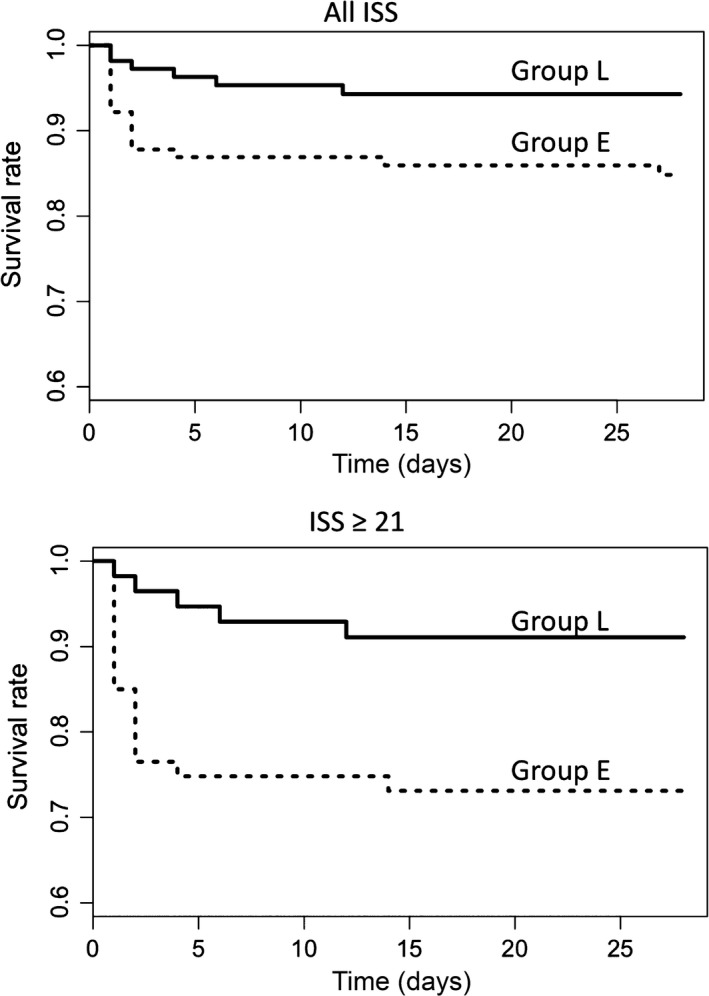
Twenty‐eight‐day survival curves of pelvic fracture patients compared between the treatment groups. Broken lines represent the curve of Group E (those hospitalized before April 2013, before the revision of the institution's massive transfusion protocol) and solid lines represent that of Group L (those hospitalized in April 2013 and later, when the massive transfusion protocol included early off‐label administration of fibrinogen concentrate). The curves of the patients with all Injury Severity Scores (ISS) (top), and those of patients with ISS ≥ 21 (bottom) are shown.

Figure 2 the shows 28‐day survival curves of the patients compared between the groups. The log–rank test gave χ^2^‐values of 5.2 (*P* = 0.022) and 6.7 (*P* = 0.009) for 1 degree of freedom in the patients with all ISS and ISS ≥ 21. The number of the 28‐day mortalities (rate) were 17 (0.15) and 6 (0.06) in Group E and Group L, respectively. The relative risks of 28‐day mortality between the groups were 0.37 (0.15–0.91) and 0.33 (0.13–0.84) in patients with all ISS and ISS ≥ 21, respectively. The results indicated significant discrepancies of the survival curves between the groups, and that the discrepancy was greater in patients with ISS ≥ 21.

## Discussion

The intergroup comparison confirmed that the characteristics, injury severity, and status on admission were homogenous among the groups (Table 1). There was no significant difference in the number of transfusions and implementation of the interventions between the groups (Table 2), and the impact of the revision on those treatments remained unclear.

The multivariate analysis using Cox's proportional hazard model identified the revision of MTP and low ISS as the independent factors significantly related to improved survival, but not RTS (Table 3). Information on physical status on admission was missing in a considerable number of patients (Table 1). They were mainly patients transferred by helicopter, and were likely to show defective values. Thus, these missing values possibly resulted in overestimation of the RTS and Ps. Consequently, the impact of RTS on survival may be underestimated by the multivariate analysis. The univariate analyses in the stratified patients also revealed that the patients with severe trauma (ISS ≥ 21) benefitted more from the revision.

As the final substrate of coagulation and the ligand of platelet GPIIb/IIIa receptors, fibrinogen plays a key role in clot formation. Fibrinogen is also the first coagulation factor to fall below a critical level during development of coagulopathy after massive hemorrhage.^4^ Hypofibrinogenemia results not only from hemodilution by fluid supplementation to maintain blood pressure, but also from fibrinogenolysis due to hyperfibrinolysis induced by tissue‐type plasminogen activator released from injured endothelial cells.^11^ In this context, fibrinogen is the key molecule in trauma‐induced coagulopathy to be targeted for supplementation.^12^ Thus, early correction of hypofibrinogenemia would be a promising approach to prevent deadly coagulopathy and mortality after massive traumatic hemorrhage.

In contrast with fresh frozen plasma, the efficacy of fibrinogen concentrate in the recovery of plasma fibrinogen levels and subsequent hemostasis was reported in hereditary and acquired hypofibrinogenemia,^13^ including those derived from trauma.^14^, ^15^ Encouraged by the volume of pharmacovigilance data showing the safety profile for fibrinogen concentrate,^16^ off‐label use of the concentrate in Japan to treat massive hemorrhage during aortic surgery^17^ and obstetrical emergencies has been reported.^18^ Based on these scientific and clinical findings, we revised our MTP to include early administration of fibrinogen concentrate in April 2013.

Surgical hemostasis is less efficient and unreliable in controlling hemorrhage associated with pelvic fractures, compared to that associated with other traumas.^19^, ^20^ Therefore, survival of pelvic fracture patients depends on hemostasis by TAE and control of coagulopathy by early correction of impaired coagulation factor levels.^21^, ^22^, ^23^ Otherwise, incomplete hemostasis and persistent coagulopathy results in devastating hemorrhage and short‐term mortality, especially in the patients with severe multiple trauma.^24^ In this context, the results of the study are reasonable and conform with our clinical experience.

A major limitation of the study derives from the substantial change in threshold and timing for administration of the fibrinogen concentrate to the patients in Group L during the study period (Fig. 1). Disunity in the administration criterion within the group prevented exact evaluation of the impact on the survival by the revision of MTP. Another major limitation derives from the absence of a clear objective criterion for activation of MTP throughout the study period. The activation was left to the clinical decision, and its consistency among the groups was not guaranteed. In the same context, consistency for the implementation of surgical or radiological interventions was not guaranteed. The possible bias in the activation of MTP and the implementation of interventions may influence the discrepancy of the survival between the groups. Because of these limitations, the results were insufficient to attribute the improved survival in Group L totally to the revision of MTP.

These limitations of the study warrant further investigation with an increased number of the patients treated with objective and consistent criteria for the activation of MTP and the administration of fibrinogen concentrate.

## Conclusion

The revision of the institutional MTP to include aggressive off‐label administration of fibrinogen concentrate was related to improved short‐term outcomes of pelvic fracture patients. The severely injured patients with high ISS benefitted more from the revision. However, due to limitations of the study, the improvement could not be attributed totally to the revision.

## Conflict of interest

None Declared.
